# Physiological Chemistry

**Published:** 1898-10

**Authors:** John A. Miller


### Progress in Medical Science.

### PHYSIOLOGICAL CHEMISTRY.

By JOHN A. MILLER, Ph. D.

### ABSORPTION OF OXYGEN BY THE LUNGS.

JOHN S. HALDANE and J. Lorrain Smith (*J. Physiol.*, 1897; *Jour. Chem. Soc.*, 1898). The absorption of oxygen in the lungs cannot be explained by diffusion alone, as the normal oxygen tension in the blood is higher than in the alveolar air, and in some animals higher than in the atmosphere. Fall of body temperatures caused a marked fall in this tension. Increase of oxygen in the alveolar air causes an almost proportional increase in the oxygen tension of the arterial blood. Diminution of the oxygen tension in the alveolar air causes a fall in that of the blood; but want of oxygen, whether produced by carbonic oxide poisoning, by diminution of atmospheric pressure, or of percentage of oxygen in the air, causes a marked increase in the relative excess of arterial over alveolar oxygen tension. Hence want of oxygen acts as a stimulus to absorption of oxygen. The symptoms caused by diminution of the oxygen tension of the air breathed are due to fall in the oxygen tension reached by the blood in the lungs, and not to diminution in the quantity of oxygen carried by the blood from the lungs.

### METABOLISM DURING INANITION.

Daiber (*Chem. Centr.*, 1898, *Jour. Chem. Soc.*, 1898). Observations made on Succi during a twenty days' fast showed that the body weight sank about 490 grams daily, the excretion of the chlorides in the urine fell to 1 or 1.5 per cent. of the normal; chlorides were not found in the urine on the twentieth day. At the beginning of the fast, urobilin was abundant, indicating decomposition of the red blood corpuscles. The metabolism of proteid, as indicated by the discharge of urea, was very regular.

### BREAKING UP OF FAT IN THE ALIMENTARY CANAL.

Vaughan Harley (*Proc. Roy. Soc.*, 1897; *Jour. Chem. Soc.*, 1898). The absorption of milk-fat in normal dogs is compared with that in those from which the pancreas had been removed; the difference in the amount of fat left in different portions of the alimentary canal is not so great as would have been anticipated. Hydrolysis of fats into fatty acids and the glycerine occurs in the stomach, as also does saponification; this is greater in dogs without a pancreas, and is probably explained by the fact that in these animals the expulsion of fat into the intestine is delayed.

---

CEREBRO-SPINAL FLUID.

E. Nawratski (*Zeit. Physiol. Chem.*, 1897; *Jour. Chem. Soc.*, 1898.) Previous workers on cerebro-spinal fluid have all but unanimously stated that the reducing substance contained in it is not sugar. In the present case, large quantities of the fluid were obtained from calves and horses and also from men; the author thus regards his work as being more trustworthy than that of others, who mostly worked with small quantities. Although he was not successful in separating the sugar in the crystalline form, he states that otherwise the reducing substance gives all the reactions characteristic of dextrose. He did not find catechol, as Halliburton did. In the fluids he examined, globulin was the only proteid present, proteoses and peptones being absent. The sugar in cerebro-spinal fluid disappears soon after death.

---

BEHAVIOR OF COMPOUNDS OF SALICYLIC ACID IN THE ORGANISM.

Stanislas Bondznyski (*Chem. Centr.*, 1898; *Jour. Chem. Soc.*, 1898). After the use of sodium salicylate, 97.5 per cent. of it was found in the urine as salicyluric acid; after the use of ethylic salicylate, 91.3 per cent. was found in the urine after ethylenic salicylate, 47.6 per cent. was found in the urine as salicyluric acid, and 19.5 per cent. in the feces as salicylic acid. Of salicylglyceride, 86.7 per cent. passes unchanged through the alimentary canal, and 8.7 per cent. is excreted in the urine as salicyluric acid. Dichlorhydrin-salicylate appears chiefly in the urine (92.7 per cent.) After the use of salicyl compounds soluble in water, such as salicylamide, there was complete absorption, none being found in the feces.

---

PHYSIOLOGICAL ACTION OF NICOULINE.

E. Boinet (*Compt. Rend. Soc. Biol.*, 1896; *Jour. Chem. Soc.*, 1898). *Nicouline*, $C_3 H_4 O$, a colorless, inodorous substance, crystallising in rhomboidal tablets, was extracted by Geoffroy from *Robinia* nicon aublet, a leguminous plant used by the natives of Guiana to stupefy and capture fish.

The action of nicouline is on the central nervous system, especially on the bulb; after a phase of excitation, stupor sets in, the muscles are relaxed, sensation is in abeyance and the temperature falls. It is rapidly eliminated. The fatal dose for mammals is 1 milligram per 10 grams of the body weight.

---

PHYSIOLOGICAL ACTION OF COPPER.

ARNOLD KOLDEWEY (*Dissert. Berlin.*, *Jour. Chem. Soc.*, 1898.) Although it is advisable to obviate admixture of copper with the food, no noteworthy evil results follow small doses of copper, or even large doses in people in good health, or in animals that vomit readily; long continuance in the use of copper, however, produces slight degenerative changes in the liver and kidney which can only be detected on microscopical examination.

---

URIC ACID IN THE SALIVA IN THE URIC ACID DIATHESIS.

BOUCHERON (*Compt. Rend. Soc. Biol.*, 1896; *Jour. Chem. Soc.*, 1898). By the murexide test, uric acid can be detected in the saliva in patients suffering from the uric acid diathesis, particularly in the intervals between meals. An analogy is drawn between this and the occurrence of sugar in the urine in diabetes.

---

PHYSIOLOGICAL ACTION OF NAPHTHALENE.

THEO. KLINGMAN (*Virchow's Archives*, 1897; *Jour. Chem. Soc.*, 1898). A number of previous workers have noticed that the most marked effect of the administration of naphthalene is an opacity of the lens and other degenerative changes in the eye-ball. In the present research on rabbits, naphthalene dissolved in liquid paraffin was given by the stomach; in the urine, phenol was found corresponding to about a tenth of the naphthalene given. The animals were killed at varying intervals; in the early stages an irido-cyclitis was found, which is believed to be the starting point of the subsequent eye-changes in the cornea, lens and retina. The primary lesion is not in the retina, as Panas considered.

---

VEGETATION WITH AND WITHOUT ARGON.

TH. SCHLOESING, JUN. (*Compt. Rend.*, 1897; *Jour. Chem. Soc.*, 1898). Oats and feather-grass respectively were grown in confined volumes of ordinary air and air deprived of argon, the $CO_2 \setminus O_2$ being determined in each case and the general appearance, behavior, and the like, of the plants observed. The conclusion drawn from the experiment is that argon has no appreciable influence on vegetation.

### ACTIVE ABSORPTION OF OXYGEN BY THE LUNGS.

J. LORRAIN SMITH (*J. Physiol.*, 1898; *Jour. Chem. Soc.*, 1898.) Various pathological conditions (fever, toxic agents of bacterial origin, local changes in the lung produced by high pressure oxygen) interfere with the active absorption of the oxygen of the pulmonary epithelium and reduce the oxygen tension of the arterial blood to about that of the alveolar air.

